# Dropout rate and associated factors of community-based health insurance beneficiaries in Ethiopia: a systematic review and meta-analysis

**DOI:** 10.1186/s12889-023-17351-7

**Published:** 2023-12-05

**Authors:** Husien Nurahmed Toleha, Ewunetie Mekashaw Bayked

**Affiliations:** Department of Pharmacy, College of Medicine and Health sciences (CMHS), Wollo University, Dessie, 1145 Ethiopia

**Keywords:** Dropout rate, Renewal Rate, Community-based Health Insurance, Associated factors

## Abstract

**Background:**

Ethiopia aims to achieve universal healthcare using health insurance. To do so, it has been implementing community-based health insurance since 2011. However, the retention of members by the scheme has not yet been evaluated nationally. The systematic review and meta-analysis aimed to evaluate the dropout rate and associated factors among the scheme’s beneficiaries in Ethiopia.

**Methods:**

On December 19, 2022, searches were conducted in Scopus, Hinari, PubMed, Semantic Scholar, and Google Scholar. Searches were also conducted on the general web and electronic repositories, including the Ethiopian Health Insurance Service, the International Institute for Primary Health Care-Ethiopia, and various higher education institutions. The Joanna Briggs Institute’s tools and the “preferred reporting items for systematic reviews and meta-analyses 2020 statement” were used to evaluate bias and frame the review, respectively. Data were analyzed using Stata 17 and RevMan 5. To assess heterogeneity, we conducted subgroup analysis and used a random model to calculate odds ratios with a p value less than 0.05 and a 95% CI.

**Results:**

In total, 14 articles were included in the qualitative synthesis, of which 12 were selected for the quantitative analysis. The pooled estimate revealed that the dropout rate of beneficiaries from the scheme was 34.0% (95% CI: 23-44%), provided that the renewal rate was 66.0%, and was found to be influenced by socio-demographic, health status, length of enrolment, knowledge, attitude, the scheme, and health service-related variables. The southern and Oromia regions reported the lowest and highest dropout rates, with 27.0% (95% CI: 24-29%) and 48.0% (95% CI: 18-78%), respectively. The dropout rates increased from 12.3% in 2012–2015 to 34.4% in 2020–2021.

**Conclusion:**

More than one-third of the scheme’s beneficiaries were found to have dropped out, and this has been found to increase over time, dictating that a community-based strategy and intervention, from the supply, insurer, and demand sides, seem indispensable in minimizing this huge dropout rate.

**Supplementary Information:**

The online version contains supplementary material available at 10.1186/s12889-023-17351-7.

## Introduction

Universal health coverage (UHC) primarily aims to ensure that everyone has access to high-quality health care without having to pay catastrophic health-care costs [1]. However, since healthcare financing (HCF) is a major issue in low- and middle-income countries (LMICs), achieving this goal in these nations has proven more difficult. More than 50% of people living in these nations have been pushed into extreme poverty due to catastrophic healthcare costs. Because their primary method of payment for health services is direct out-of-pocket (OOP) payment [2].

Most developing countries, including Ethiopia, have committed to achieving UHC by using health insurance systems as a risk-sharing mechanism [3]. In doing so, Ethiopia launched a community-based health insurance (CBHI) program in June 2011 [4] and began to scale it up in 2015 [5] to cover the poor, unemployed, and primarily those living in deprived rural areas and provide equitable health care [6]. It was operational in 700 woredas and cities in 2019/20, covering nearly one-third of Ethiopia’s population [7].

The sustainability of CBHI schemes is determined by the growth rate, coverage ratio, and renewal rate of members. The coverage ratio measures the number of participants from the scheme’s target population, and the renewal rate measures the number of insured members who renew their membership after their coverage term expires [8]. A high renewal rate and increased membership contribute to the development of a stable insurance system capable of producing adequate funding for health care [9, 10].

The CBHI program faces significant challenges due to low enrollment and a high dropout rate, making it unsustainable [2, 11, 12]. Evidence showed that CBHI membership coverage has not had a significant impact on UHC, despite new enrollees [13]. This is because enrollment in CBHI is based on voluntary consent [6]. Voluntary health insurance schemes are characterized by high dropout rates, which is a major issue in developing countries. In India, for example, a drop-out rate of 63% has been documented [13]. In Ghana, the dropout rate increased from 34.8% in 2012 [14] to 53% in 2016 [8]. In Uganda, approximately 25.1% of households leave the voluntary CBHI scheme [15]. Similarly, one year after the implementation of CBHI in pilot districts, the dropout rate in Ethiopia increased from 18% [11] to 31.9% [16] and 37.3% [17] with later studies.

The population coverage of the scheme was inconsistent with the enrollment rate. This could be due to the fact that voluntary membership allows families to join and leave based on their health status. This leads to low participation and excludes the poorest households. Consequently, wealthier households are more likely to enroll in the scheme than those from poorer groups [18]. In turn, a high dropout rate results in adverse selection. As a result, vulnerable groups are more likely to stay active. This puts the scheme’s financial viability in jeopardy [19]. In such cases, health insurance plans will fail to improve access to care and protect members from catastrophic health costs [20]. The dropout rate is not only due to the voluntary nature of the scheme; rather, it is also known to be influenced by several other factors too [2, 17, 21].

However, in Ethiopia, to the best of our knowledge, there was no national data showing the dropout and membership renewal rates of CBHI in a nationwide situation. On the other hand, policymakers require information to maintain the CBHI scheme by identifying the sources of dropouts. Recognizing the factors that contribute to CBHI dropout may help improve program sustainability. The aim of this systematic review and meta-analysis was, thus, to provide information on Ethiopia’s CBHI membership dropout rate and its associated factors. In particular, it was to answer two basic questions: What was the extent of the CBHI membership dropout rate in Ethiopia? What were the factors influencing the membership dropout?

## Methods

### Registration and protocol

The protocol of this review was registered with PROSPERO (ID: CRD42023392567), which is available at: https://www.crd.york.ac.uk/prospero/display_record.php?ID=CRD42023392567. The framework for this review was the “Preferred Reporting Items for Systematic Reviews and Meta-Analyses (PRISMA) 2020 Statement: An Updated Guideline for Reporting Systematic Reviews” [22]; Additional File 1.

### Eligibility criteria

We included cross-sectional, case-control, and mixed study designs. Studies conducted on the CBHI scheme’s membership dropout and renewal in Ethiopia, conducted in English from 2012 onwards, both published and unpublished, were included. The included studies were chosen based on study design, area, study’s year, sample size, response rate, and main outcome. Studies with incomplete information and a high risk of bias were excluded. If a study reported the same result in more than one journal, it was considered a duplication, and only the published one with the title of interest was considered for review [16, 23].

### Information sources and search strategy

Manual and database searches were conducted to find information sources. On January 19, 2023, database searches were conducted in Scopus, Research4Life (Hinari), PubMed, Semantic Scholar, and Google Scholar. Manual searches were conducted on PubMed, Hinari, and Google Scholar. Studies from Scopus, Semantic Scholar, and Google Scholar were searched using the “Perish or Publish” database searching tool, version 8 [24]. Text words and indexed terms such as “community-based health insurance,“ “dropout,“ “renewal,“ “factors,“ and “Ethiopia” were used to search databases (Additional File 2). We also searched other sources, such as the general web and electronic repositories such as the Ethiopian Health Insurance Service (EHIS), the International Institute for Primary Health Care-Ethiopia (IPHCE), and higher institutions.

### Selection process

Duplicates and irrelevant studies were excluded using Zotero Reference Manager version 6. Two reviewers, HNT and EMB, screened the included studies first by title and abstract; second, full-text evaluation was conducted independently and then collaboratively. When disagreements took place, the issues were thoroughly discussed with both reviewers face-to-face until reaching consensus.

### Data collection process and data items

A Microsoft Excel spreadsheet was used to extract the outcome variables, the population (study units), the year of study, the context, the sample size, the response rate, the dropout rate, and the associated factors. HNT and EMB extracted the data independently, compared their findings, and reached an agreement. When there were differences, the issue was thoroughly discussed with both reviewers. Furthermore, the authors of the studies have been contacted in order to gather the missing data. After the whole extraction process has been completed, each set of data was imported and checked for accuracy using a comprehensive meta-analysis software version 3.

### Study risk of bias assessment

The risk of bias was evaluated using the Joanna Briggs Institute (JBI) checklists. All studies that met the inclusion criteria were thoroughly reviewed by HNT and EMB. Sample inclusion criteria, a description of the study subjects and setting, measurement validity and reliability, confounding factors and strategies for dealing with those factors, and the appropriateness of the outcome measures were assessed for bias. The JBI critical appraisal tool included 10 and 8 items to assess case-control and cross-sectional studies, respectively. Finally, only studies with low and medium risk were included in the review. Any discrepancies in the scores were resolved through discussion.

### Effect measures

For each included study, prevalence, proportion, inverse variance, and odds ratios were calculated. The *x* squared, *p* value with a 95% confidence interval, and odds ratios were calculated for the summary effect.

### Synthesis methods

We conceptually classified the outcome variables using thematic strategies for the qualitative synthesis. Based on the qualitative synthesis, preliminary computations of effect measures such as prevalence and proportion, as well as odds ratios of CBHI dropout, were performed using a Microsoft Excel spreadsheet. Subgroup analyses were performed to compare the effect estimates on the outcome variables across studies based on regions and the year of the studies.

The pooled proportion of the dropout rate was calculated using Stata version 17, while the odds ratios (ORs), to determine the strength of the ratio between the dropout rate and the non-dropout rate or renewal rate (event vs. non-event), were calculated using a random method in RevMan 5.4.1. A p-value less than 0.05 with a 95% CI was used to determine the level of overall statistical significance, including heterogeneity.

### Reporting bias and certainty assessment

The reporting bias was evaluated by looking at whether or not the studies were published if they had more than one version, published or unpublished. It was also investigated by the studies and the years of publication of the studies. The authors of the studies with incomplete or missing data have been contacted. Studies with incomplete data were excluded.

The *I*^*2*^ statistic was used to assess heterogeneity between studies, with the following thresholds: 0–40% might not be important; 30–60% indicates moderate heterogeneity; 50–90% suggests substantial heterogeneity; and 75–100% represents considerable heterogeneity [25]. Inverse variance (percentage of weight) was used to calculate each study’s impact on the overall meta-analysis. The funnel plot was used to visually ascertain the possibility of publication bias. Sensitivity analysis was performed by unchecking studies with small sample sizes, though the heterogeneity remained almost the same.

## Results

### Study selection

**Fig. 1:**
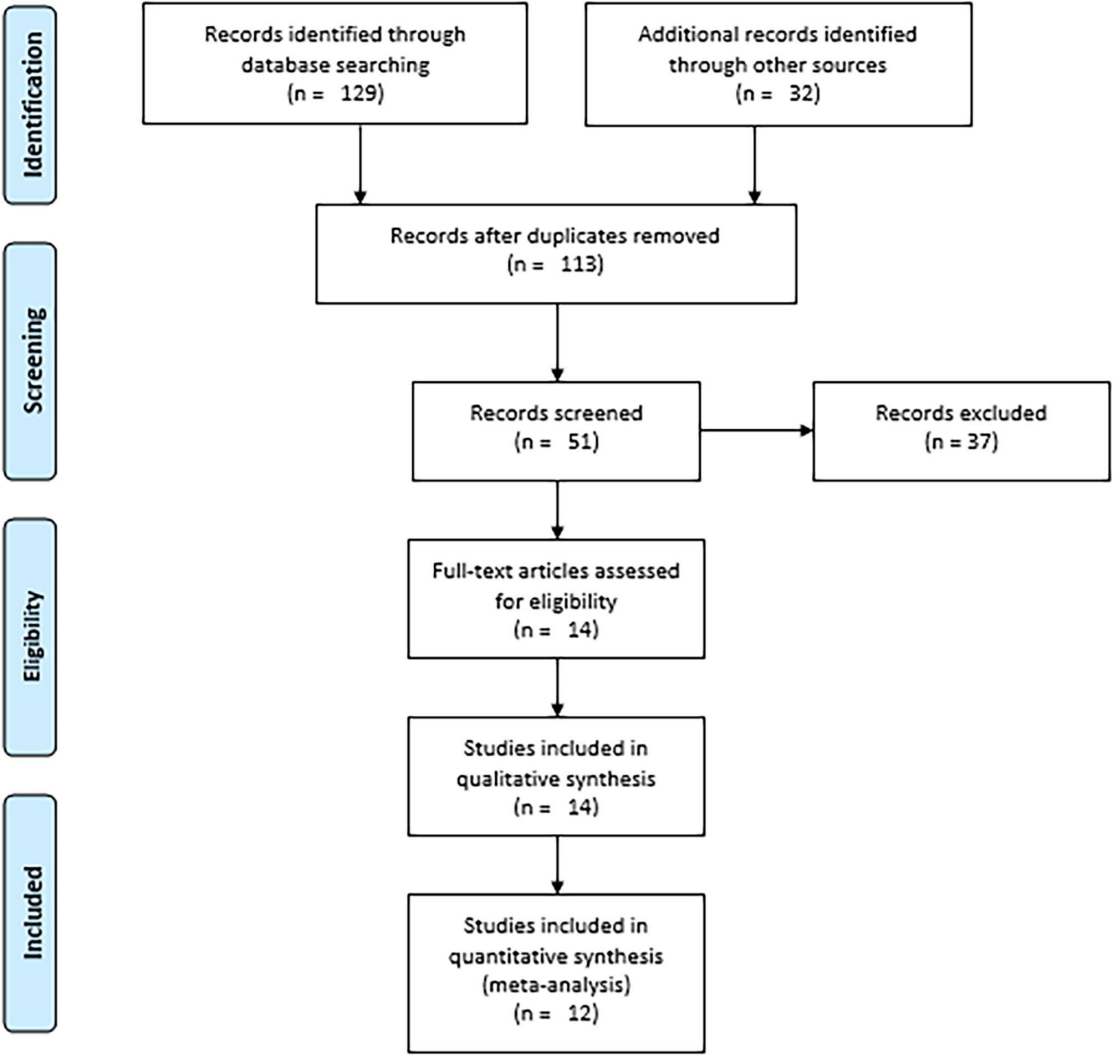
PRISMA flow diagram showing the selection processes of the included studies

A total of 161 resources were identified (Fig. 1). Databases were used to identify 129 of them: Hinari (n = 14), PubMed (n = 12), Scopus (n = 1), Google Scholar (n = 56), and Semantic Scholar (n = 46). The remaining 32 sources were obtained from other sources, such as websites (n = 15), organizations (n = 5), and citation searching (n = 12). After duplicates were removed (n = 48), 113 records were found. After excluding 62 studies based on relevance, 51 records were screened for title and abstract evaluation. Following the review of the title and abstract, 14 records were selected for full text evaluation. With the full-text article evaluation, all of them met the inclusion criteria. Finally, 14 studies were included in the systematic review.

### Study characteristics

The Amhara (n = 5), Oromia (n = 3), and SNNPR (n = 3) regions accounted for roughly 78.5% (n = 11) of the total studies included in the systematic review. The other studies were conducted in Addis Ababa (n = 1) and in the national context (n = 2). The total sample population of all included studies was 8,179, of which 8,038 (98.3%) were found to be actual participants. Table 1 summarizes the results of the individual study characteristics.

**Table 1:** Characteristics of the individual included studies, Ethiopia (n = 14)

Study ID	Design	Area	Year	SS	RR	Main Outcome
Gashaw et al., 2022 [28]	Mixed-CS	AA	2021	634	626	Renewal rate
Hussien et al., 2022 [29]	Cross-sectional	Amhara	2020	1257	1232	Adherence to CBHI Scheme
Ashagrie et al., 2020 [17]	Cross-sectional	Amhara	2020	584	584	Dropout rate
Workneh et al., 2017 [4]	Cross-sectional	Amhara	2015	530	511	Compliance to CBHI
Asmamaw, 2018 [9]	Mixed-CS	Amhara	2017	810	810	CBHI membership renewal
Wassie et al., 2023 [31]	Case-control	Amhara	2018	634	634	Determinants of dropout
Mebratie et al., 2015 [11]	Mixed-CS	National	2012-13	489	483	Dropout rate
Tefera et al., 2021 [27]	Mixed-CS	National	2019	336*	336	CBHI with quality Service
Kebite, 2020 [26]	Cross-sectional	Oromia	2020	624	584	Dropout rate
Mekuria et al., 2020 [33]	Cross-sectional	Oromia	2018	195	195	Dropout rate
Eseta et al., 2020 [16]	Cross-sectional	Oromia	2020	634	617	Dropout rate
Kaso et al., 2022 [32]	Cross-sectional	SNNPR	2021	551	537	Renewal rate
Zepre et al., 2022 [6]	Mixed-CC	SNNPR	2021	525	513	Factors for dropout
Worku, 2019 [30]	Mixed-CC	SNNPR	2018	376	376	Factors for dropout

**Note.** *=only CBHI enrolled members from the total sample, SNNPR: Southern Nations, Nationalities and Peoples Region; AA = Addis Ababa, RR: Response Rate; SS: Sample Size; CC = Case Control; CS = Cross Sectional

### Risk of bias in studies

**Fig. 2:**
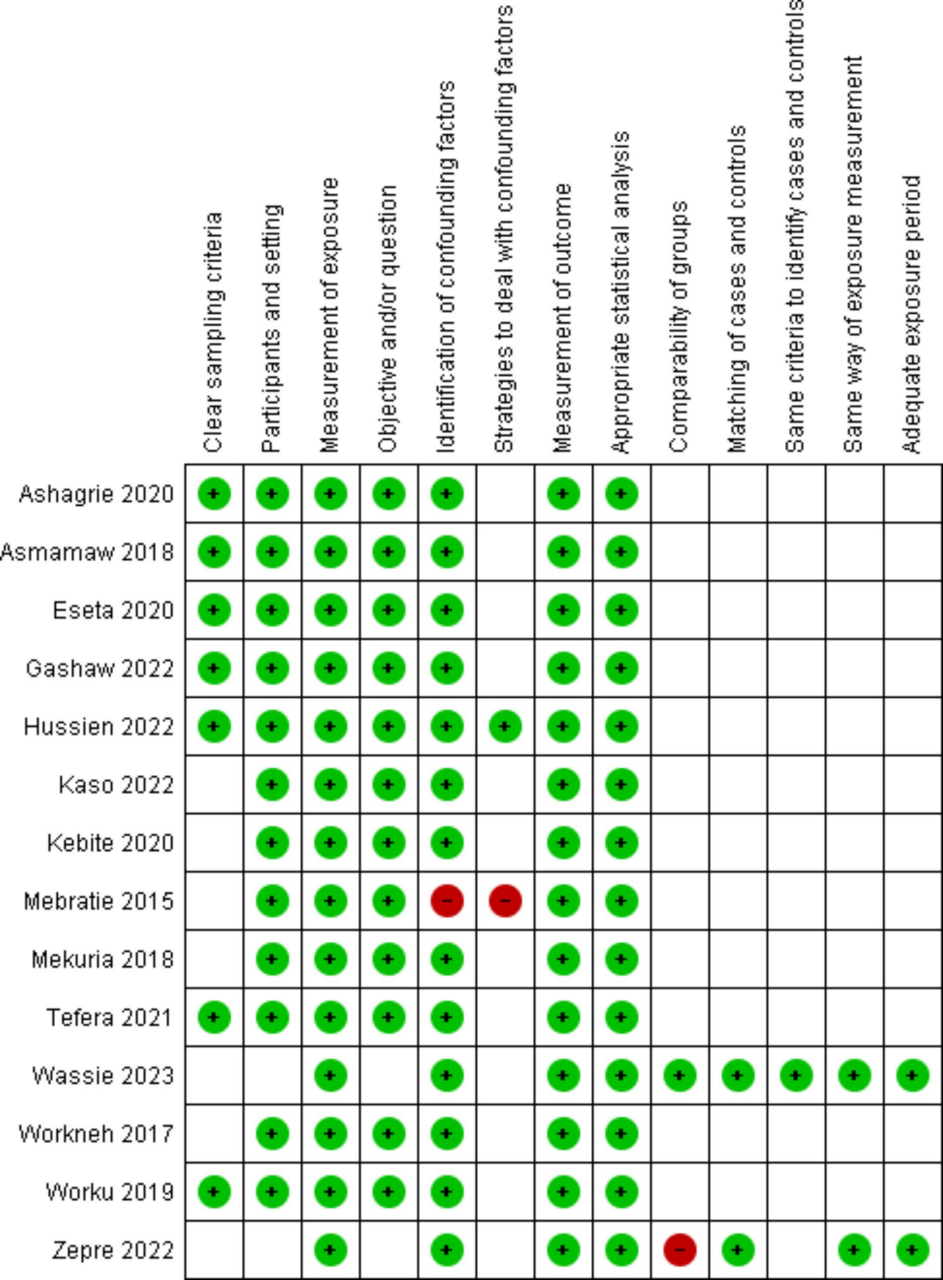
Summary of the risk of bias in the included studies; Green: low risk, Red: high risk, Unmarked: unclear risk

Based on the JBI’s critical appraisal tools, they were used to assess the risk of bias for the included studies. Accordingly, for pure cross-sectional studies and mixed studies with a cross-sectional design, scores of 7 and above were labeled as low risk, 5–6 as medium risk, and 4 and below as high risk. In the case-control studies, scores of 6 and below, 7–8, and 9–10 were rated as high, medium, and low risk, respectively (Table 2). Figure 2 summarizes the results of all included studies’ risk of bias assessments.

**Table 2:** Assessments results of risk of bias for each included study

Study ID	Score		Risk
	Tally	Percentage	
Hussien et al., 2022 [29]	8/8	100.0	Low
Kebite, 2020 [26]	6/8	75.0	Medium
Mekuria et al., 2020 [33]	6/8	75.0	Medium
Ashagrie et al., 2020 [17]	7/8	87.5	Low
Eseta et al., 2020 [16]	7/8	87.5	Low
Kaso et al., 2022 [32]	6/8	75.0	Medium
Mebratie et al., 2015 [11]	5/8	62.5	Medium
Gashaw et al., 2022 [28]	7/8	87.5	Low
Workneh et al., 2017 [4]	7/8	87.5	Low
Asmamaw, 2018 [9]	7/8	87.5	Low
Wassie et al., 2023 [31]	9/10	90.0	Low
Tefera et al., 2021 [27]	7/8	87.5	Low
Zepre et al., 2022 [6]	7/10	70.0	Medium
Worku, 2019 [30]	7/8	87.5	Low

### Results of individual studies

#### Qualitative results

**Fig. 3:**
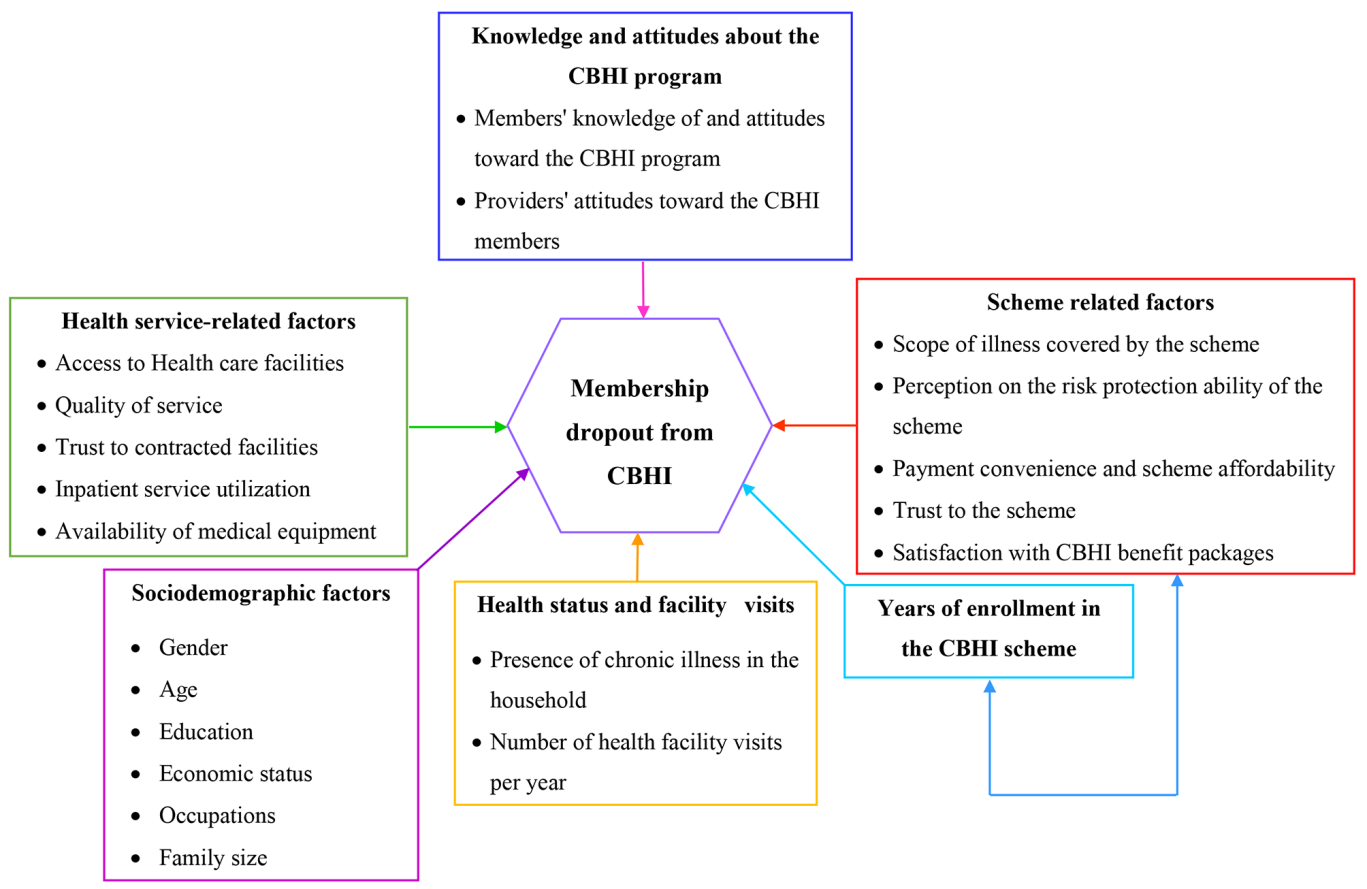
The relationship between the determinants of it and the CBHI membership dropout in Ethiopia

The review identified six major themes that influence CBHI dropout, as shown in Fig. 3. socio-demographic factors, health supplier- or facility-related factors, scheme-related factors, knowledge and attitude toward the CBHI scheme, years of enrollment, and health status Based on quantitative investigations, Table 3 provides an overview of the direction of influence of the variables determining the dropout rate from CBHI scheme.

**Table 3:** Summary of the direction of influence of the factors determining the dropout rate from the CBHI scheme in Ethiopia

Factors	(+)	(-)	Total	Summary
**Sociodemographic factors**				
Gender (male)	2	-	2	Positive
Age (older)	1	1	2	Inconclusive
Education (higher)	2	-	2	Positive
Economic status (high)	4	-	4	Positive
Occupations (merchant)	2	-	2	Positive
Family size (large)	-	4	4	Inverse
**Health service-related factors**				
Access to Health care facilities (lack)	3	-	3	Positive
Quality of service (low)	8	-	8	Positive
Trust to contracted facilities (low)	3	-	3	Positive
Inpatient service utilization (lack)	1	-	1	Positive
Availability of supplies (shortage)	2	-	2	Positive
**Scheme related factors**				
Benefit packages & scope (limited)	4	-	4	Positive
Perception on the risk protection ability of the scheme (poor)	1	-	1	Positive
Payment convenience and scheme affordability (positive perception)	-	3	3	Negative
Trust to the scheme (low)	5	-	5	Positive
Satisfaction with CBHI benefit packages (low)	3	-	3	Positive
**Knowledge and attitudes about the CBHI program**				
Members’ knowledge of and attitudes toward the CBHI program (low)	7	-	7	Positive
Providers’ attitudes toward the CBHI members (negative)	1	-	1	Positive
**Years of enrollment in the CBHI scheme (long)**	-	5	5	Negative
**Health status and facility visits**				
History of chronic illness in the household (yes)	-	5	5	Negative
Number of facilities visits per year (frequent)	1	1	2	Inconclusive

#### Theme 1: socio-demographic factors

Age, sex, occupation, financial position, level of education, and household size were found to be important socio-demographic factors influencing CBHI membership dropout. The respondent’s age has a mixed effect on decisions for CBHI renewal. One study found that older household heads were less likely to drop out of the CBHI scheme [16]. The other one, however, revealed that older members were less likely to adhere to CBHI program requirements [4]. Concerning gender, men are positively associated with dropping out of the scheme [26], whereas women are more likely to renew membership [9, 26]. The CBHI dropout was influenced by education. Two of the studies found that dropping out of the CBHI program was more likely for those with higher education levels [16, 27]. In relation to family size, four of the studies found that families with many family members were more likely to renew their membership in the program or were less likely to drop out [16, 26, 28, 29]. Based on their economic position, households with the highest income had a higher likelihood of dropping out of the program [6, 26, 30], while those from the poorest groups were more likely to stay with the program [11]. Regarding occupation, two studies revealed that, compared to farmers, merchants were more likely to drop CBHI programs [4, 30].

#### Theme 2: health supplier or facility-related factors

The review found that about 70% of the included studies reported that participants’ views on factors related to health facility services were the main determinants of whether they renewed or dropped their membership in the CBHI scheme. Regarding access, three of the studies reported that facility access [17, 26, 30] and a lack of supplies like drugs and medical equipment [6, 28] were important factors in the dropout rate of the CBHI program. The other one stated that households were more likely to renew their membership when they received inpatient care through CBHI coverage [29]. Eight of the examined studies reported that service quality was inversely associated with CBHI membership dropout. Six of them indicated that dropping out of the CBHI program was positively correlated with respondents’ low perceptions of the quality of the service [6, 16, 26, 28, 29, 31]. The other two studies showed that people were more willing to renew their CBHI membership when they thought the quality of healthcare services was high [9, 32]. Moreover, trust in contracted health facilities substantially predicted readiness to renew membership [9] and had an inverse relationship with CBHI dropout [16, 29].

#### Theme 3: CBHI scheme-related factors

Payment convenience, scheme affordability, levels of satisfaction, benefit packages, risk protection ability of the scheme, trust in the scheme, and scope of illness covered by the scheme were identified as factors relating to the CBHI program that affect the CBHI membership dropout rate. Three of the studies found that the CBHI program’s dropout rate was significantly influenced by low levels of satisfaction and limited benefit packages [16, 28, 30]. Renewal of membership was influenced by the ease of making payments and the perceived affordability of the program [28, 30, 31]. Households with a positive perception of the program’s risk protection ability were less likely to drop out [29], but dropout was more likely if OOP costs for stock-out services were not reimbursed [31]. Additionally, a limited range of illnesses covered by the program is positively associated with participants dropping their membership [16, 26]. Moreover, five of the studies highlighted the importance of program trust in minimizing program dropout or maintaining CBHI membership renewal [6, 9, 16, 28, 29].

#### Theme 4: knowledge and attitude about the CBHI scheme

Knowledge of the CBHI system and attitudes toward the CBHI system were found to be the determinants of dropout rates in seven of the studies that were reviewed. Four of them indicated that a limited knowledge of the risk-sharing principles of the CBHI program raised the dropout rate [4, 11, 17, 31]. The other three revealed a positive association between respondents’ program knowledge and CBHI membership renewal [17, 28, 32]. Positive attitudes toward the CBHI program reduced dropout rates [4, 6, 32]. Besides, the likelihood of dropout was enhanced by providers’ adverse attitudes toward CBHI members [16].

#### Theme 5: years of enrollment

The length of enrolment was found to be an important factor that is substantially associated to CBHI dropout in five of the reviewed studies. As members continue to enroll for a longer period of time, the likelihood of dropping out decreases [6, 17, 26, 28, 32].

#### Theme 6: health status

Health status of household members and the frequency of their medical visits were found to be major predictors of continuing CBHI membership in seven of the review studies. Four of them indicated that the existence of a chronic illness in the household affected their decision to continue participating in the program [11, 29, 33] and positively associated with maintaining CBHI membership [28]. The other two studies showed that households without a history of chronic illness had higher dropout rates [31, 33]. In terms of health visits, households without a history of frequent hospital visits were more likely to drop out than their counterparts [6]. The result of the other study, however, showed that people who visited frequently had a higher likelihood of leaving their CBHI program [17].

#### Quantitative results

The pooled analysis includes 6,982 participants from the 12 included studies, with 44.93%, 19.99%, 13.03%, 8.97%, and 13.08% from Amhara, Oromia, National, Addis Ababa, and SNNPR, respectively (Table 4).

**Table 4:** Dropout rate of the population group, Ethiopia (n = 12)

Study ID	Participants	Events	Prevalence	Region
Gashaw et al., 2022 [28]	626	205	32.80	Addis Ababa
Hussien et al., 2022 [29]	1232	359	29.10	Amhara
Ashagrie et al., 2020 [17]	584	218	37.30	Amhara
Workneh et al., 2017 [4]	511	40	7.83	Amhara
Asmamaw, 2018 [9]	810	294	36.30	Amhara
Mebratie et al., 2015 [11]	574	106	18.47	National
Tefera et al., 2021 [27]	336	104	30.95	National
Kebite, 2020 [26]	584	436	74.70	Oromia
Mekuria et al., 2020 [33]	195	74	37.90	Oromia
Eseta et al., 2020 [16]	617	197	31.90	Oromia
Kaso et al., 2022 [32]	537	93	17.30	SNNPR
Worku, 2019 [30]	376	188	50.00	SNNPR

The overall dropout rate of beneficiaries from the scheme was determined to be 34.0% (95% CI: 23-44%), given a renewal rate of 66.0%. As depicted in Table 5, a regional subgroup analysis revealed varying dropout rates: 22.0% (95% CI: 19-25%) at the national level, 28.0% (95% CI: 13-42%) in Amhara, 48.0% (95% CI: 18-78%) in Oromia, 27.0% (95% CI: 24-29%) in SNNPR, and 33.0% (95% CI: 29-37%) in Addis Ababa.

**Table 5:** The sub-group analysis of the proportion of the dropout rate from CBHI in Ethiopia by region (n = 12)

Region		Proportion (95% CI)	Weight (%)
**National**	Mebratie et al., 2015 [11]	0.18 [0.16, 0.22]	8.37
	Tefera et al., 2021 [27]	0.31 [0.26, 0.36]	8.28
	**Subtotal**	**0.22 [0.19, 0.25]**	**16.65**
**Amhara**	Workneh et al., 2017 [4]	0.08 [0.06, 0.10]	8.40
	Asmamaw, 2018 [9]	0.36 [0.33, 0.40]	8.37
	Ashagrie et al., 2020 [17]	0.29 [0.27, 0.32]	8.40
	Hussien et al., 2022 [29]	0.56 [0.52, 0.60]	7.70
	**Subtotal**	**0.28 [0.13, 0.42]**	**33.50**
**Oromia**	Mekuria et al., 2018 [33]	0.38 [0.31, 0.45]	8.14
	Kebite, 2020 [26]	0.75 [0.71, 0.78]	8.36
	Eseta et al., 2020 [16]	0.32 [0.28, 0.36]	8.35
	**Subtotal**	**0.48 [0.18, 0.78]**	**24.85**
**SNNPR**	Worku, 2019 [30]	0.50 [0.45, 0.55]	8.27
	Kaso et al., 2022 [32]	0.17 [0.14, 0.21]	8.37
	**Subtotal**	**0.27 [0.24, 0.29]**	**16.64**
**Addis Ababa**	Gashaw et al., 2022 [28]	0.33 [0.29, 0.37]	8.35
**Overall**		**0.34 [0.23, 0.44]**	**100.00**

**Fig. 4:**
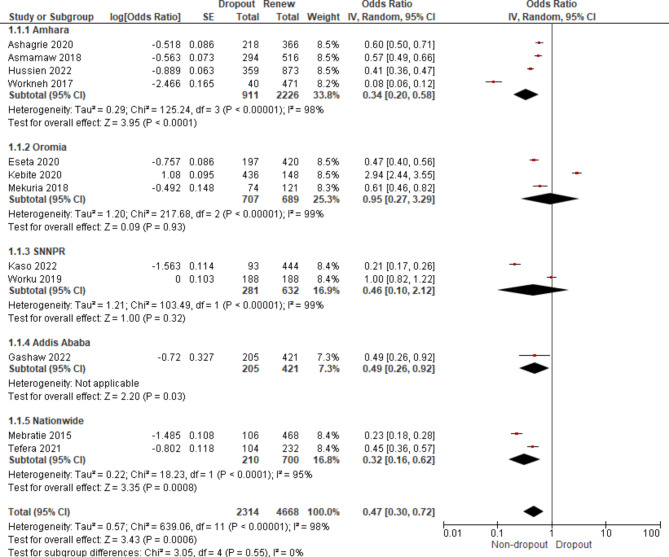
The forest plot for membership dropout rate by region

Coming to the ratio strength of the dropout rate to renewal rate, the dropout rate was found to be significant for the sub-groups: Amhara (OR = 0.34; 95% CI: 0.20, 0.58), Addis Ababa (OR = 0.49; 95% CI: 0.26, 0.92), and Nationwide (OR = 0.32; 95% CI: 0.16, 0.62). However, it was found to be not significant for Oromia (OR = 0.95; 95% CI: 0.27, 3.29) and SNNPR (OR = 0.46; 95% CI: 0.10, 2.12) (Table 6). As depicted in Table 6; Fig. 4, the pooled result showed that the membership dropout rate was found to be 47% more likely and was found to be significant (OR = 0.47; 95% CI: 0.30, 0.72).

**Table 6:** The pooled effect by region using the odds ratios

Outcome/Subgroup	Studies	Participants	Statistical Method	Effect Estimate
1. Amhara	4	3137	Odds Ratio (IV, Random, 95% CI)	0.34 [0.20, 0.58]
2. Oromia	3	1396	Odds Ratio (IV, Random, 95% CI)	0.95 [0.27, 3.29]
3. SNNPR	2	913	Odds Ratio (IV, Random, 95% CI)	0.46 [0.10, 2.12]
4. Nationwide	2	910	Odds Ratio (IV, Random, 95% CI)	0.32 [0.16, 0.62]
5. Addis Ababa	1	626	Odds Ratio (IV, Random, 95% CI)	0.49 [0.26, 0.92]
**Overall**	**12**	**6982**	Odds Ratio (IV, Random, 95% CI)	**0.47 [0.30, 0.72]**

**Fig. 5:**
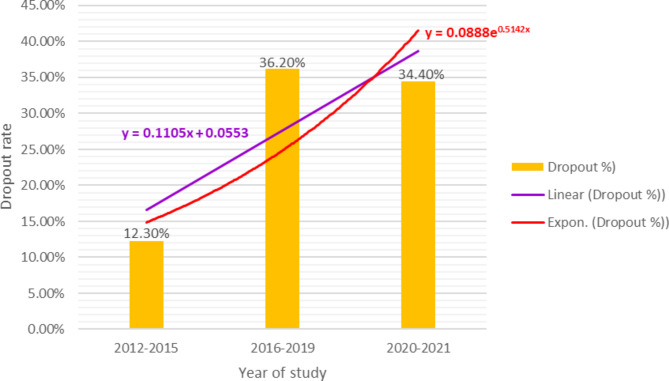
The trend of CBHI membership dropout rate in Ethiopia

**Fig. 6:**
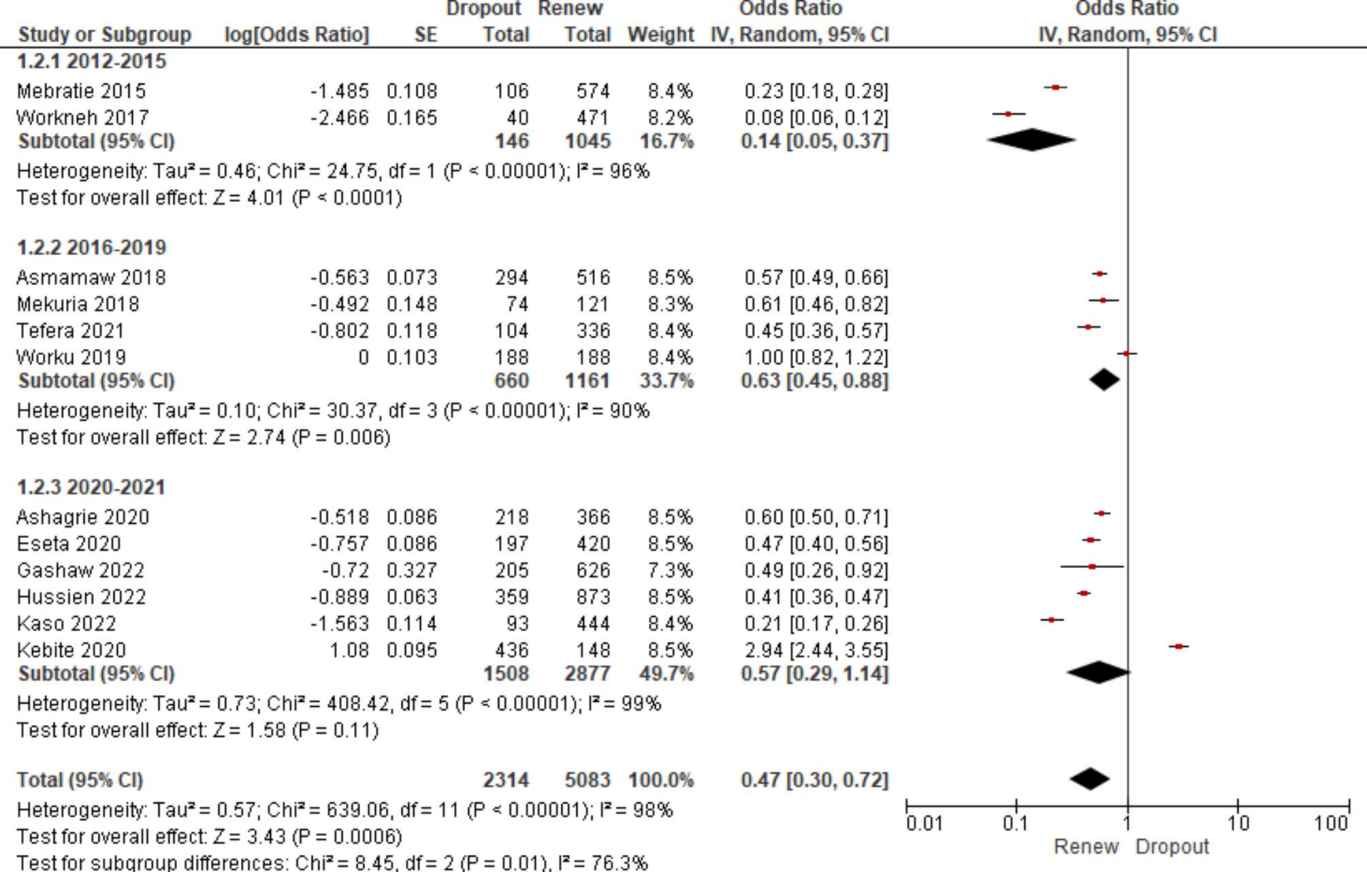
Membership dropout rate by the year of studies

The subgroup analysis by year of study showed that the dropout rate has been progressively increasing from time to time. The dropout rates from 2012 to 2015, 2016 to 2019, and 2020 to 2021 were 12.3%, 36.20%, and 34.4%, respectively (Fig. 5). Though the dropout rate from 2020 to 2021 was not found to be significant, the overall pooled result showed that the dropout rate through the rears was highly significant (OR = 0.47; 95% CI: 0.30–0.72). The odds for the dropout rate increased from 0.14 in 2012 to 0.57 in 2020 and 2021 (Fig. 6).

### Reporting biases and certainty of evidence

**Fig. 7:**
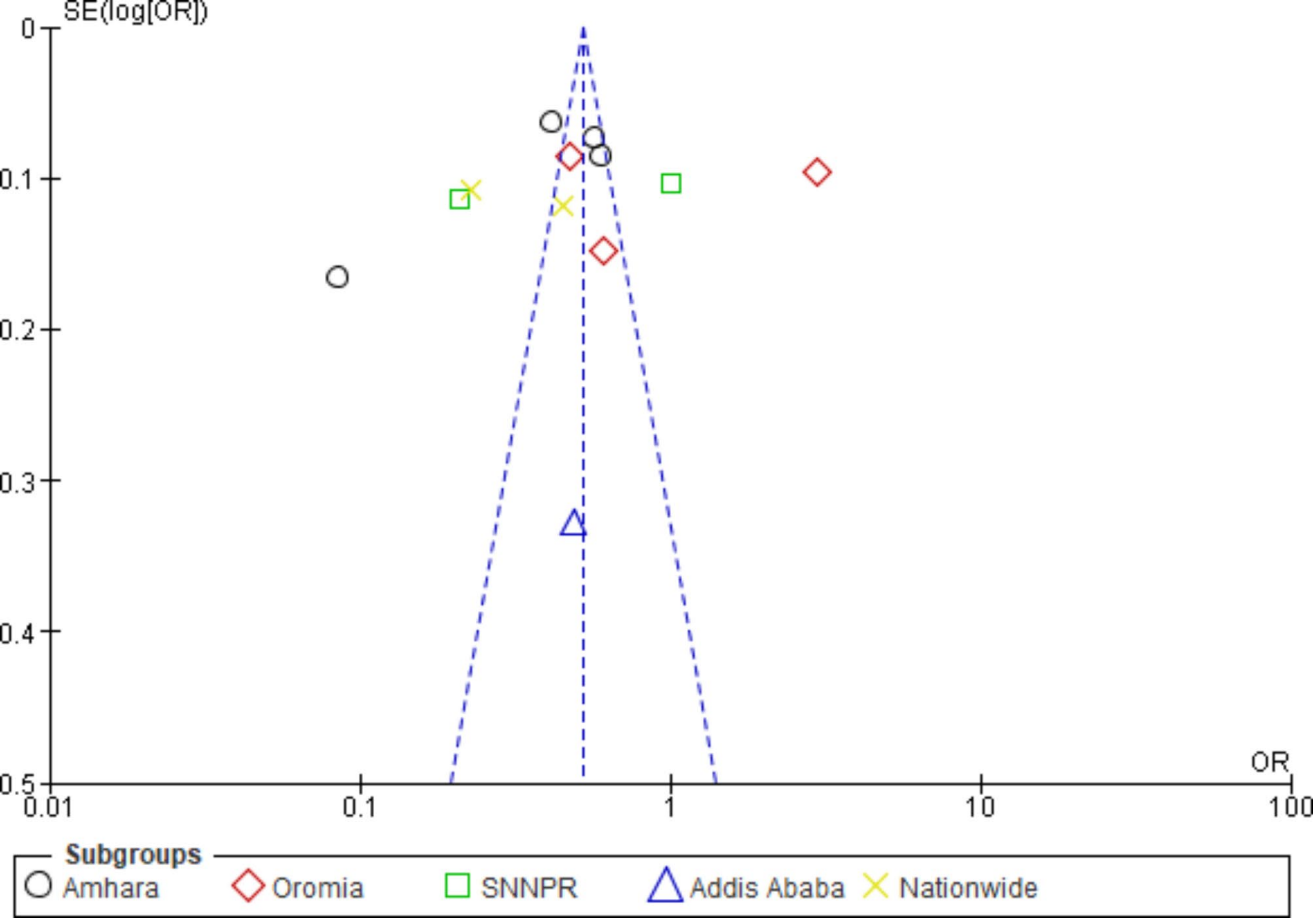
The summary analysis of publication bias

We conducted sub-group analyses based on regions and the year of the studies. Heterogeneity was evaluated with the I^2^ statistic. It (I^2^ = 0%, p = 0.55) showed no heterogeneity between regions (Fig. 4). The heterogeneity between the sub-groups by year was substantially high (I^2^ = 76.3%, p = 0.01); refer to Fig. 6. However, the overall pooled result showed that the heterogeneity was consistently high (I^2^ = 98%, p < 0.00001), indicating considerable heterogeneity [25]. As a result, we used a random-effects model with a 95% CI to pool the membership dropout rate. As shown in Fig. 7, funnel plots were also used to investigate the possibility of publication bias, but no extreme outlier has been found.

## Discussion

**Fig. 8:**
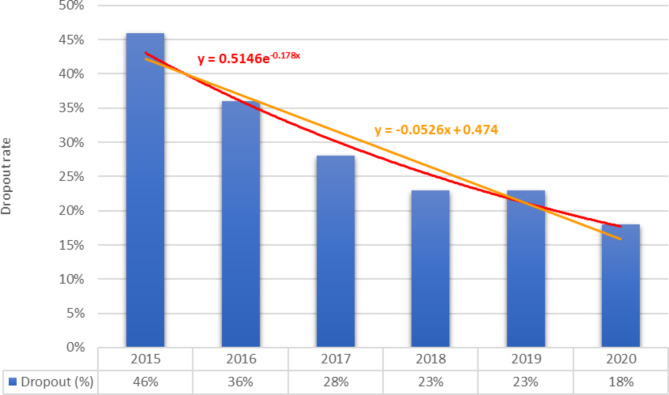
The trend of CBHI membership dropouts reported by EHIS from 2015 to 2020 [52]

CBHI is crucial for reducing the cost of catastrophic health care, improving cost recovery, expanding access to services, reducing the risk of extreme poverty, and advancing toward UHC [34]. The CBHI enrollment coverage goal of 80% by 2020, however, was challenged by dropouts from the program [35]. Consequently, the goal of this review was to identify the Ethiopian CBHI drop-out rate and its contributing factors. A review found that 34.0% (95% CI: 23-44%) of respondents had dropped out of the program, i.e., the proportion of the renewal rate was 66.0%. The proportion of the dropout rate has been found to be varied by region in that the lowest was in SNNPR, which was 27.0% (95% CI: 24-29%), while the highest was in Oromia region, 48.0% (95% CI: 18-78%). The dropout rate has also been found to be increasing over time, which, as depicted in Fig. 8, is in contrast to the report by EHIS [7].

The lowest dropout rate in SNNPR and the highest dropout rate in Oromia might seem paradoxical when considering the actual scenario. This is because the design characteristics of the scheme in Oromia are better than those in SNNPR. CBHI members in SNNPR have limited access to tertiary healthcare services; insured households can use tertiary services only at the nearest public hospitals, whereas those in Oromia may access care from public hospitals both within and outside the region. Households insured in SNNPR cannot claim reimbursements for using healthcare services from private providers if medical equipment or drugs are unavailable at CBHI-linked facilities [18]. The disparity might be due to inadequate information, poor cooperation between institutions and insurers, and substandard healthcare services in Oromia, as reported by a study in Nepal [36].

The dropout rate in our review was almost equivalent to the reports of other studies conducted in Ghana (34.8%) [14] and Burkina Faso (30.9%) [2]. However, far greater dropout rates in Burkina Faso (45.7%) [2], Senegal (72.6%) [12], and India (67.3%) [37] and lower dropout rates in Vietnam (21.1%) [38] were reported. The variation could be attributed to the various sociodemographic traits of study participants, as well as the study period and locations.

The findings of this review show that age, gender, financial situation, level of education, and household size are major socio-demographic determinants impacting CBHI membership dropout. In terms of gender, women are more likely to renew their membership [9, 26]. This aligns with the findings of an Indian investigation [13]. This may be because women tend to be more risk-averse than men. The review found that age had a conflicting impact on dropout. One of the reviewed studies, which was supported by data from Vietnam [38], revealed that older individuals had a lower dropout rate from CBHI [16], whereas another study, which was supported by data from Nigeria [39], revealed that older individuals were less likely to renew their CBHI membership [4]. This could be explained by contextual differences among the studies, wherein in one situation, older members may be more prone to disease and concerned about what would happen to their family if they became sick, but in another context, they may be economically and socially indecisive. Regarding education, compared to households that couldn’t read and write, those with higher education were more likely to leave the CBHI [16, 27]. One possible explanation is that educated people comprehend health insurance benefit packages, operating principles, and risk-sharing systems more easily, as well as the uncertain nature of health crises and their effects, which enhances their inclination for risk aversion. In terms of household size, larger family members were less likely to drop out of the CBHI system [16, 26, 28, 29], which was supported by data in rural India [40]. This could be because households with larger families want to retain their insurance coverage to limit the danger of financial failure. This gives policymakers ideas for enacting policies to promote small families in order to address the issue of adverse selection. In terms of income, poor households were more likely to stay in the scheme [6, 11, 26, 30]. This could be linked to social assistance and benefits such as fee waivers for the poor. This may lead to adverse selection.

Dropout rates were greatly influenced by health-related factors. Hospital access, in-patient treatment coverage, trust, and quality services are all critical health-related factors in the CBHI program’s dropout rate. Household members who did not have access to a hospital were more likely to cancel their CBHI schemes [11, 17, 26, 30]. In addition to the medical expense, secondary costs of illness such as transportation, food, bed, and other opportunity costs when seeking health care could explain the increased risk of dropout. Furthermore, a lack of resources, such as medications, was substantially linked to CBHI dropout [6, 28]. In contrast to the findings of an Indian study [13], receiving inpatient care through CBHI coverage motivates households to renew their membership [29]. This disparity could be explained in terms of service quality and income disparities among the study participants.

In terms of service quality, willingness to renew membership was significantly connected to the perceived quality of health care [9]. Poor healthcare quality increased dropout rates from the scheme [6, 16, 26, 28, 29, 31]. This was comparable to research undertaken in Burkina Faso, Sudan, and Senegal [2, 12, 41]. A study in southern Ethiopia found that perceived high-quality health-care services were associated with a greater CBHI membership renewal rate [32]. Similar findings were reported from Ghana [14], Rwanda [42], Burkina Faso, and Senegal [2, 12]. This means that providing high-quality services is essential for the CBHI scheme’s success because it influences patients’ perceived value and pleasure. Unless scheme participants receive high-quality health care, they may lose trust in the program and acquire a negative attitude toward it. Dropout from CBHI was strongly linked to trust in contracted health facilities [16, 29] and could strongly determine membership renewal willingness [9]. This is consistent with research findings from Cambodia [43], Burkina Faso [2], and Senegal [12], which revealed that poor public health services contribute to low trust, resulting in low CBHI membership renewal.

Dropouts from the CBHI plan have been linked to scheme-related characteristics such as benefit packages, premium affordability, trust, and the scheme’s ability to defend against risk. Poor satisfaction and restricted benefit packages have been identified as important determinants of dropout from the CBHI scheme [16, 28, 30]. This was in line with previous findings [14, 21]. The perception of the scheme’s ability to protect against risk and the cost of premiums were major factors in membership renewal [28, 29, 31]. One of the fundamental goals of universal health coverage is to eliminate the need to pay for health-care services directly [1]. It is possible to achieve this by raising adequate funds through prepayment modes and pooling approaches to deliver equity health services [34]. Trust in the scheme was critical in lowering scheme dropout and preserving CBHI membership renewal [6, 9, 16, 28, 29, 33]. Similar findings were reported from Senegal [12] and Cambodia [43]. A meta-analysis and systematic review findings from LMIC also revealed a similar report [21].

Dropout rates from the scheme are affected by knowledge and attitude. Poor understanding of the CBHI program’s risk-sharing concepts increases the program’s dropout rate [4, 11, 17, 31]. This was supported by reports from Senegal [12], Burkina Faso [2], and Tanzania [44]. Similar to the findings from Uganda [45], this review revealed that membership renewal was positively associated with respondents’ knowledge and attitude toward the CBHI program [4, 6, 28, 32]. As in Ghana and Benin [46, 47], the review found that unfavorable provider attitudes toward CBHI members increased the likelihood of dropout [16]. This could be because members are unsatisfied with the providers’ differential care based on patients’ socioeconomic position. As a consequence, rather than renewing their membership, they decide to explore alternative health risk coping measures. Health practitioners and government officials should collaborate to bridge the knowledge gap in the community by spreading information about the CBHI benefit package.

Another key factor influencing responders’ CBHI program membership renewal is the length of engagement [6, 17, 28, 32]. This finding is supported by research conducted in India [13] and Ethiopia [48, 49]. Long-term CBHI participants can help increase knowledge and awareness of the CBHI program, which reduces dropout rates. Additionally, when family members remain in the scheme for an extended period of time, they may consider the money they have placed [13].

Furthermore, the health status of household members strongly predicts sustained CBHI membership [9, 11, 28, 29]. A household with chronic illness was less likely to drop out [11, 29, 31, 33] and was strongly related to CBHI membership renewal [28]. Similar findings were reported from Sudan [41] and Ghana [14]. Members who are ill are more likely to renew their membership in order to avoid risk. It has two major implications. One, CBHI promotes access to health care for high-risk individuals by avoiding catastrophic health costs or the risk of complications if they refuse treatment due to financial inability. Second, the scheme’s long-term viability and performance may be called into question [50]. The frequency of health facility visits was associated with the dropout rate [17]. In contrast, households that had no prior experience with frequent visits were more likely to drop out [6]. This disparity could be attributed to patient-provider controversy, members’ trust in a health professional and contracted health facility, and the financial catastrophe they face.

To summarize, some of the review’s findings are consistent with other countries’ investigations, while others contradict them. The observed disparity could be explained by the study environment, which includes the study population, fee waiver policies, and benefit packages offered by the scheme.

### Policy and practical implications

UHC, as rooted in the Sustainable Development Goals (SDGs), aims to provide individuals, families, and communities with health security and access to essential care services without financial hardship, thereby enabling a transition to more productive and equitable societies and economies [51]. The CBHI’s high dropout rate makes meeting UHC objectives difficult. The authors recommend that the EHIS improve the scheme’s benefits package while service providers and managers improve the quality of health services, engage, empower, and boost members’ confidence and trust. The districts’ offices of the CBHI scheme should reimburse payments on time and pay attention to the socioeconomic characteristics of their members. Furthermore, health education should be provided to increase participants’ knowledge and perception of the CBHI program’s benefit packages and risk-sharing principles.

### Limitations

The association between the dependent variable (dropout rate) and the independent variables could not be determined due to the diverse reports from the included studies. Only English-language papers were included. Furthermore, not all regions of Ethiopia were included in the review, as the studies considered were conducted only in Oromia, Amhara, SNNPR, and Addis Ababa.

### Direction to future research

A comprehensive national study employing both qualitative and quantitative methods is essential to examine the extent and causes of CBHI dropout among households. Additionally, conducting regular customer satisfaction surveys is crucial, along with revising premium fee levels based on reimbursement to members for OOP expenses incurred due to referrals.

## Conclusion

In Ethiopia, more than one-third of CBHI members dropped their membership. Age, gender, occupation, socioeconomic status, level of education, and household size have all been identified as important socio-demographic factors influencing dropout rates. Dropout from the CBHI scheme was positively associated with supply side factors such as hospital inaccessibility, shortage of supplies, lack of satisfaction with the service, limited benefits packages, low perception of the scheme’s risk protection ability, and limited scope of illness covered by the scheme, as well as demand side factors such as poor perceived quality of service, members’ negative attitude toward CBHI members, and providers’ negative attitude toward CBHI members. Furthermore, payment convenience, scheme affordability, good knowledge, receiving inpatient care through CBHI coverage, trust in contracted health facilities, and the CBHI scheme all contributed to a lower scheme dropout rate.

### Electronic supplementary material

Below is the link to the electronic supplementary material.

Supplementary Material 1

Supplementary Material 2

## Data Availability

The data that support the findings of this study are available within the article.
