# Supplementary material for: Dropout rate and associated factors of community-based health insurance beneficiaries in Ethiopia: a systematic review and meta-analysis
Source: BMC Public Health. 2023 Dec 5;23:2425. doi: 10.1186/s12889-023-17351-7 (PMC10698931; doi:10.1186/s12889-023-17351-7)
Supplement: Supplementary file 2 — Supplementary Material 2 [file 12889_2023_17351_MOESM2_ESM.docx]

# **Supplementary file 2: Database search strategy**

1. **PubMed (n=12)**

Search: **(((Dropout) OR (Renew)) AND (community-based health insurance)) AND (Ethiopia)** Filters: **Abstract, from 2012/1/1 - 2023/1/1**

(("dropout"[All Fields] OR "dropouts"[All Fields] OR ("renew"[All Fields] OR "renewal"[All Fields] OR "renewals"[All Fields] OR "renewed"[All Fields] OR "renewing"[All Fields] OR "renews"[All Fields])) AND ("community based health insurance"[MeSH Terms] OR ("community based"[All Fields] AND "health"[All Fields] AND "insurance"[All Fields]) OR "community based health insurance"[All Fields] OR ("community"[All Fields] AND "based"[All Fields] AND "health"[All Fields] AND "insurance"[All Fields]) OR "community based health insurance"[All Fields]) AND ("ethiopia"[MeSH Terms] OR "ethiopia"[All Fields] OR "ethiopia s"[All Fields])) AND ((fha[Filter]) AND (2012/1/1:2023/1/1[pdat]))

**Translations**

**Dropout:** "dropout"[All Fields] OR "dropouts"[All Fields]

**Renew:** "renew"[All Fields] OR "renewal"[All Fields] OR "renewals"[All Fields] OR "renewed"[All Fields] OR "renewing"[All Fields] OR "renews"[All Fields]

**community based health insurance:** "community-based health insurance"[MeSH Terms] OR ("community-based"[All Fields] AND "health"[All Fields] AND "insurance"[All Fields]) OR "community-based health insurance"[All Fields] OR ("community"[All Fields] AND "based"[All Fields] AND "health"[All Fields] AND "insurance"[All Fields]) OR "community based health insurance"[All Fields]

**Ethiopia:** "ethiopia"[MeSH Terms] OR "ethiopia"[All Fields] OR "ethiopia's"[All Fields]

1. **Research4Life (R4L) - Hinari (n=14)**

Search: ((Dropout) OR (renew)) AND (Community based health insurance) AND (Ethiopia)

Selected by:

Date of publication: from 2012/1/1 - 2022/08/27

Content Type: Any type

Discipline: Any type

Language: English

Limit to: Items with full text online

1. **Advanced Google Scholar (n=56)-using “Perish or Publish” software**
2. Search: renew or dropout "community-based health insurance" Ethiopia
3. Year: 2012-2023
4. **Semantic scholar using perish or publish (n=46)- using “Perish or Publish” software**

Search key words: Dropout renew community-based health insurance Ethiopia

Year==2012-2023

1. **Scopus search (n=1)- using “Perish or Publish” software**

Search key words: Dropout renew community-based health insurance Ethiopia

Year==2012-2023
